# Editorial

**Published:** 1871-02

**Authors:** 


### Editorial.

### *Illustrations of Quackery.*

To the Editors of the Chicago Medical Journal:

*Gentlemen*—I enclose an advertisement of the "Chicago Medical and Surgical Institute" from the *Western Home*, a periodical published in your city, in which R. A. Gunn, M.D., "assisted by the best surgeons in Chicago," solicits "consultations by letter, with enclosures of $2, and professes his readiness to perform "all kinds of surgical operations" "*very moderate* in all cases." The said periodical also contains a "Medical Department" conducted by the same "R. A. Gunn, M.D., Professor of Surgery, Bennett Medical College," in which, at the end of two columns of ignorant charlatanry, he warns the public against a traveling competitor, and assures his readers that Bennett Medical College "discountenances all forms of quackery."

Pray, who is this Gunn that makes so loud a report with such small charges? Is he a "great Gunn" or only an "Ancient Pistol"? If the latter, cannot he be made to go off without puffs?

Respectfully yours,

A Minor Canon.

New York, Jan. 1, 1871.

In reply to the above, it is only necessary to state that the "Chicago Medical and Surgical Institute" is an offshoot of the *Bennett* (!!) *Medical College*, an "Eclectic" concern which vegetates in a cockloft over some warehouse or commission store in this city.

The caution which the advertiser puts forth against some fancied competitor is simply stupendous in its impudence. It is the old *stop-thief* cry over again.

It is only necessary to advise *distant* readers that Prof. Moses Gunn of Rush Medical College, for many years previously Prof. of Surgery in the University of Michigan, and well known throughout the United States, is in nowise responsible for the pranks of the person who wears, temporarily at least, his surname. This is an old dodge that our New York correspondent has certainly had occasion to see something of at home.

While we are on this subject we may as well say to many correspondents who have sent us copies of handbills, newspaper advertisements, etc., of all sorts, issued by persons claiming to be graduates of respectable medical colleges, and to have some wonderful methods of cure, etc., etc., *ad nauseam*, that, whatever the previous *status*, these parties in so doing directly cut themselves off from the legitimate profession, and must accept the situation assigned them by gentlemanly instinct as well as the spirit and letter of the code. From Du Quoin, Ill., comes, for instance, the announcement of an "Infirmary," duly illustrated with startling wood-cuts, and yet, if we are not mistaken, one or both the "Superintendents" are graduates of the Apostolic Reform School of this city. These gentlemen say they have treated in their rookery "THIRTY THOUSAND PATIENTS, including many strange and terrible cases." And to their statements plentiful certificates are not wanting.

Under these circumstances all medical men who wish to sustain a reputable character should forego mere clamor, or lachrymose appeals to the legislature for "protection," but each for himself secure that solid basis of sound learning, large and cultivated experience, and clear mental apprehension, which will commend him, and the profession he represents, to the confidence of all educated and thinking minds.

### *The U. S. Marine Hospital.*

E. C. Rogers, M.D., has resigned the surgeoncy of the Marine Hospital, to take effect on the 1st inst. It is but simple justice to him to state that for the several years he has been in charge, the duties have been discharged with a fidelity and ability which have received the highest commendation from the official inspectors, and the general applause of all visitors. Everything about the institution has moved like clockwork, patients have received the most excellent care and treatment, and yet with that economy of expenditure which the Government needs everywhere exhibited, but, unfortunately, rarely finds illustrated.

His successor, N. T. QUALES, M.D., is a graduate of Rush Medical College, and a gentleman of excellent qualifications, both professional and personal. He has had considerable experience in the charge of hospitals in this city, and we have no doubt will fill the position with credit to himself and satisfactorily to the Government.

### *Responsibility.*

It seems necessary to repeat frequently that all communications to this JOURNAL, and all selections inserted, must rest entirely on their merits for reception by readers. Some we may personally agree with, and others not at all. How can progress take place unless the varied observations and conclusions of intelligent men be advanced and compared? We believe in the largest liberty of disagreement consistent with a sincere search for the truth. Yet, while going thus far, and even admitting that in the wildest forms of human opinion there are always elements of truth present which may render them deserving the notice of the true medical philosopher, we must be permitted to state, here, that upon several important theoretical and practical points we differ *ioto cœlo* from several valued correspondents. An early opportunity will be taken to outline our own views upon these important matters.

For all articles whose authorship is not indicated by source, name, *nom de plume*, or initials, the Senior Editor is solely responsible.

These explanations have been given, again and again, nevertheless almost daily we receive letters asking us whether we endorse certain particular communications. We endorse nothing but good faith.

### *The American Practitioner.*

The editors of this excellent new monthly have laid us under especial obligation by presentation of two elegantly bound volumes, comprising the numbers for 1870. The paper, typography and binding are superb, and we are happy to add, the matter contained is fully worthy its beautiful dress. Some of the best articles of the last year originally appeared in the *Practitioner*. It aims to be especially a journal of therapeutics, on the plan of its English namesake. Published at Louisville, Ky., by John P. Morton & Co. Edited by Professors Yandell & Parvin. $3 a year.

***Protection.***

Another of the biennial attempts to secure legislation for the protection of the profession against quackery, etc., etc., is now in progress at the State capitol. Will not our uneasy brethren overhaul their primary reading, and study the fable of the frogs who prayed for a king?

The only protection the profession want from the legislature is, that they promptly pass the bill now before them, a copy of which is printed on another page of this No. of the JOURNAL.

***American Journal of Obstetrics—Diseases of Women and Children.***

By the courtesy of B. F. Dawson, M.D., Managing Editor and Proprietor, we receive the first two vols., handsomely bound, of this important scientific and practical record of gynæcology. It is published quarterly by W. A. Townshend & Adams, New York, and edited by Drs. Noeggerath, Dawson and Jacobi. $4.00 a year. Single numbers, $1.25.

This is the most valuable and ably conducted journal devoted to this department that it has been our fortune to meet. It richly deserves the full and generous support of the profession.

***Original Communications***

Have so taken up our pages the present No. that we are obliged to put over a large quantity of material already prepared. Will our correspondents please to recollect that, to insure prompt appearance of their favors, they should reach us on or before the tenth of the month preceding date of publication?

***A BILL To Promote the Science of Medicine and Surgery in the State of Illinois.***

SECTION 1. It shall be lawful in cities and counties whose population exceeds twenty thousand inhabitants, for superintendents of penitentiaries, wardens of poor-houses, coroners, and city undertakers, to deliver to the professors and teachers in medical colleges and schools in this State, and for professors and teachers to receive, the remains or body of any deceased person, for purposes of medical and surgical study; *provided*, that said remains shall not have been regularly interred, and shall not have been desired for interment by any relative or friend of said deceased, within twenty-four hours after death; *provided, also*, that the remains of no person who may be known to have relatives or friends shall be so delivered or received without the consent of said relatives or friends; *and provided*, that the remains of no one detained for debt, or as a witness, or on suspicion of crime, or of any traveler, or of any person who shall have expressed a desire in his or her last sickness that his or her body may be interred, shall be delivered or received as aforesaid, but shall be buried in the usual manner; *and provided, also*, that, in case the remains of any person so delivered or received shall be subsequently claimed by any surviving relative or friend, they shall be given up to said relative or friend for interment.

SEC. 2. And it shall be the duty of the said professors and teachers decently to bury, in some public cemetery, the remains of all bodies after they shall have answered the purposes of study aforesaid; and for any neglect or violation of the provisions of this act, the party so neglecting shall forfeit and pay a penalty of not less than twenty-five nor more than fifty dollars, to be sued for by the health officers of said cities, or other places, for the benefit of their department.

SEC. 3. The remains or bodies of said persons as may be so received by the professors and teachers, as aforesaid, shall be used for the purposes of medical and surgical study alone, and in this State only; and whoever shall use such remains for any other purpose, or shall remove such remains beyond the limits of this State, or in any manner traffic in the same, shall be deemed guilty of a misdemeanor, and shall, on conviction, be imprisoned for a term not exceeding one year in a county jail.

SEC. 4. Every person who shall deliver up the remains of any deceased person in violation of, or contrary to, any or all of the provisions contained in the first section of this act, and every person who shall receive said remains, knowing the same to have been delivered contrary to any of the provisions of said section, shall each and every of them be deemed guilty of a misdemeanor, and shall, on conviction, be imprisoned for a term not exceeding two years in a county jail.

